# A Case of *Rabies Canina*, with the Appearances on Dissection

**Published:** 1817-10

**Authors:** R. Webster

**Affiliations:** Member of the Royal College of Surgeons in London, and Surgeon of the 51st Regiment of Light Infantry


*A Case of* RABIES CANINA, *with the Appearances on Dissection.* By R. WEBSTER, Member of the Royal College of Surgeons in London, and Surgeon of the 51st Regiment of Light Infantry.

IT hath fallen to my lot to add another example to the list of the fatal effects of the poison of a rabid animal, when introduced into the human body.

Fully prepared as I was, by the descriptions which I had read of this disease; and although there did not appear a single symptom or circumstance which I might not have anticipated; yet every shade and feature of the picture was so truly melancholy, that I could not but experience feelings of the most unpleasant and mortifying nature, and such as, I imagine, must be excited in the mind of every man who has the misfortune to behold a human being suffering under the agonizing torture of hydrophobic spasms, especially when he is convinced that he has not the power of rendering the smallest relief or assistance to the unhappy victim!

Fortunately, Rabies is a rare disease; and it probably seldom happens that a practitioner sees more than one or two cases in the course of many years experience.* The present was the first that either I, or seven other gentle-

---

* "Hydrophobiam ex morsu animalis rabiosi nunquam vidi."—*Heberden.*

men who attended with me, had seen; yet the disease was so strongly characterised, that a momentary observation of the wretched subject assured us that there could be no mistake as to its nature.

There are, I understand, sceptics who do not believe that the human constitution is capable of being affected by this disease. I can assure them, that, beyond the possibility of a doubt, it is; and that most of the descriptions which have been given of it are true, as far as regards its general features. Language, however, is inadequate to paint it in all its horrors: to do this, would require that hand which produced the wonderful picture of the Transfiguration.

About half past eleven o'clock of the morning of the 20th of August, 1817, I went, in company with Mr. Bradley, Surgeon in the Royal Artillery, and Mr. Fitzpatrick, one of my assistants, to visit Francis Brown, a labourer, who, while at work on the 21st day of July last, was attacked by a strange dog, which had hid himself amongst some timber. On our way to the house, I learned that he had been bitten in the hand; that it had become painful, and that the pain was extending up his arm.

From reflections made long ago, I had determined, that if I should be called to a patient who had been bitten by a rabid animal, and in whom pain had commenced in the wounded part, I would amputate the limb, as the only possible chance of averting the threatened mischief: strongly impressed with this idea, I entered the room. We found the man sitting up in his bed, supported by his wife: his countenance was pale, and expressive of great anxiety; he was sobbing greatly, and the muscles of the fore part of his neck were convulsed. He cast his eyes upon us, but instantly turned them to the window, which was a little open. This paroxysm, it appeared, was excited by his having swallowed some castor oil; and was continued by the cool air blowing in upon him. We requested him to take a little liquid; which he did, after many strong efforts, the very request having induced an increase of convulsive sobs. He was laid down, and the window was shut. On being questioned as to his feelings, he complained of an excessive pain in his left hand, arm, and shoulder; of difficult breathing; and of his not being able to swallow. He said that he had not been entirely free from pain since the infliction of the wound; but on Saturday it began to be severe, and had gradually extended up his arm, to the shoulder and breast. Since Tuesday noon he had not been able to move his arm; but when he wished to turn himself, he was obliged to lift it with his right hand. He said that the pain was now excruciating. On the hand there were some small cicatrices of a red colour; the largest, and what had always been the most painful, was on the integuments covering the first phalanx of the little finger.

I immediately procured such materials as the room afforded, and formed a tourniquet, which I applied and tightened, immediately below the axilla: he instantly said he was free from pain; and, in less than a minute and a half, he raised his arm nearly perpendicular to the body of his own accord, and used it with perfect freedom.* This sudden change, from excruciating pain to perfect ease, had a great effect upon him. I now directed the window to be opened: he cast his eyes quietly towards it; but he had no spasm, and said it occasioned him no inconvenience. I then gave him some liquid, but he drank it with great difficulty.

Messengers were now sent to Mr. Hennin and Dr. Johnson, requesting their immediate attendance; in the mean time, the tourniquet, which had been allowed to remain on about twenty minutes, was removed, and the vena mediana cephalica opened, from which thirty ounces of blood were drawn.

At the commencement of this operation, his pulse was 74, and intermitted at every seventh or ninth stroke. It was small, but became more open as the blood flowed. It was of a natural strength, but rather slow in the systole. His tongue was of a natural colour in the middle and at the edges; but the sides had a brown streak running along them: his gums were moist and red; but his teeth as dry as possible. The abdomen was distended and tense: he complained of some uneasiness on pressure between the scorbiculus cordis and umbilicus; but lower than this, pressure occasioned him no uneasiness.

*His left arm was lying outside of the bed-clothes; the rest of his body, up to his mouth, he covered very carefully.* He was perfectly rational, expressed some anxiety as to his fate, but the greatest dread appeared to arise from an apprehension of the window being opened. He watched every one who approached it; and if an arm was raised

---

* From this instant, to the time of his death, the pain did not return. At present I shall make no observations on this extraordinary fact, but have to beg that the experiment may be repeated in the next case of rabies which occurs.

even to put back the curtain, he was immediately seized with spasms. This dread formed one of the most prominent features in the character of the disease; and did not leave him, even to his latest moments. I counted his pulse during the flowing of the blood; it intermitted less during the loss of the last ten ounces, but kept constantly at 74. Shortly after this period, until eleven at night (the last time I saw him), it was about 64, and with less intermission than before the bleeding. He bore the loss of blood well; but at this late period of the disease, I did not think it would be prudent to push it further. He had had no stool for three days; I therefore gave him four pills, composed of the extractum colocynth. comp. cū hydr. submuriat. *Those I put into his mouth one by one, and he swallowed them without difficulty.**

I directed an enema; but as he had had a motion previous to the arrival of my orderly-man, and I was absent at the moment, it was not administered. With the stool he passed some urine, which he had not done for twelve hours before. The other gentlemen having engagements, I waited in the neighbourhood, and saw him frequently.

At half past two, I found that he had been apprised of his danger; and that this, together with the appearance of distress which his wife exhibited, had occasioned him considerable disturbance. He endeavoured, however, to appear calm; but the spasms were more violent, and were more readily excited. On my asking him if he thought he could drink? he began to sob convulsively, and he was at length so severely affected, that all appearance of the trachea was lost, the sterno-cleido-mastoidei muscles became contracted, and for the space of two inches from the clavicles nearly touched each other. He seemed highly gratified when I told him that he ought to endeavour to sleep, as he had been persuaded that he should, on no account, allow himself to fall into that state. He expressed great satisfaction, also, on being able to turn himself on his side, which he said he had not been able to do on account of the great pain † and stiffness of his arm, "until the thing was put upon it."

---

* In giving him the pills, I *unguardedly* put my finger and thumb into his mouth; had the spasms supervened, and the skin been abraded by his teeth, nothing less than deep scarification and cauterization would have satisfied me.

† At this time his wife informed me that he had some fever on Saturday night; that on Sunday he had "cold chills;" and that, from Tuesday morning, he had not been able to bear his left arm under the bed-clothes, until after the pressure was made by the artificial tourniquet.

Reasoning from the effect which the extractum belladonnæ produces on the iris, when applied to the eye-lid, I had in one of my notes asked, if its application to the throat in hydrophobia might not lessen the morbid irritability, and thereby render deglutition less painful? With this view I applied it, but without any effect.*

During the examination of his pulse, in the first instance, he kept his eyes constantly fixed upon the seals which were attached to the watch; and if, by accident, I let them touch his face, he became greatly distressed, and sobbed violently. Nothing, however, could divert his attention from the window, which he still regarded anxiously, nothwithstanding another obnoxious object was so near to him.

At half past three the following gentlemen arrived: Dr. Johnson, Messrs. Hennin, Bradley, Hughes, and Fitzpatrick. Every one seemed equally affected at the melancholy sight. He was now become so irritable, that a fly passing near him, alighting upon his face, occasioned him great distress. It was directed that he should take a quarter of a grain of the extractum belladonnæ every second hour. A blister, five inches in breadth, was applied from the base of the cranium to the sacrum, and an enema directed to be given.

At seven in the evening I found all the symptoms aggravated, with the exception that the circulation was not more hurried, the pulse still remaining at 64. His tongue was become dry, and of a brown colour throughout its whole surface. The room being warm, some one attempted to open the window, when a most violent paroxysm took place, his whole trunk appearing to participate in the convulsive motions. He had had no return of pain in his hand or arm. His thirst was very great. He was raised, and a little stewed apple put between his fingers, in order that he might convey it to his mouth; but the distress it occasioned was too great to permit him to effect it; he held it about a minute and a half, during which time his countenance alternated in expressions of determined resolution and deep despair; he at length found himself unequal to the task, and in the most piteous tones and submissive manner, begged to be permitted to decline

---

* It is obvious from the dissection, that this or any other medicine of the same class must have been useless, when organic disease to such an extent had taken place. Could I have succeeded in completely paralyzing the nerves of the fauces, it could have had no good effect.

a farther trial. His wife offered to put the apple into his mouth, but he said "she could not hit the time;" meaning the momentary relaxation of spasm.

With a feather I applied some milk and water to his lips. On its approach, the mouth became closed involuntarily; but, in a short time, he put out his tongue and licked his wet lips without the smallest inconvenience.* He had had a stool from the enema.

11. P. M. The same gentlemen who had met in the morning were present, together with Dr. Seeds and Mr. Stewart, R. N.

His thirst was now become intense, and seemed to be insufferable. His tongue was extremely dry, and of a darker colour. On some one proposing that he should try a little water, the spasms became dreadfully severe; he put his lower lip between his teeth; his eye-lids were raised; his countenance was altogether wild, fearful, and expressive of despair! Shortly afterwards he appeared to form some desperate resolution. At his request I raised him on his breech, and supported him; his shirt and bed-clothes were drenched with perspiration. His wife put a cup of currant jam into his hand: he looked upon it with horror; but summoning up resolution, he plunged his finger into it. Another pause ensued—he then, in the most desperate manner, pushed his finger as far as possible into his mouth; his teeth and lips were closed before he could withdraw it; this he twice repeated; it was a scene of perfect horror! This was the last time I saw him. I was informed that he died at six the next morning.

Two men had been appointed to sit up with him; from whom I learned the following account.

At half past eleven he took three grains of opium, and the same quantity at one. He was restless, and had no sleep; he frequently seized the bed-clothes and his own arms; he stared wildly towards the window, but knew what was said to him. He had frequent grinding of his teeth; his skin was hot, and he had an inclination to vomit. At about five o'clock he appeared greatly exhausted, and two or three times raised his legs, and again extended them; some "frothy phlegm" was discharged from his mouth, he died without a struggle.

---

* I should think from this circumstance, that if a piece of thin sponge, dipped in lemon-juice, or vinegar and water, and attached to the night-cap by pieces of tape, was allowed to remain immediately under the lower lip, it would be a likely method of relieving thirst. I regret I did not think of trying it in time.

DISSECTION, *seven Hours after Death.*

Present, Deputy-Inspector Hennin, Dr. Johnson, Dr. Seeds, Mr. Bradley, Mr. Stewart, R. N. and myself.

Nothing unusual on the external surface of the body. The arm was opened, and the vessels and nerves traced from the bite to the axilla; but, excepting a slight blush of the neurilema of one of the superficial nerves, nothing particular was discovered.

*Abdomen.* Stomach inflated with gas; its internal surface greatly inflamed, and even abraded near the cardiac orifice. The same might be said of various portions of the small intestines, whose peritoneal coverings were, in many places, highly injured and inflamed. Almost the whole of the colon, excepting the lower portion of the sigmoid flexure, was singularly contracted, and in some points apparently strictured.

*Thorax.* The right lung was gorged, and somewhat inflamed. The pericardium contained more water than natural, but neither it nor the heart shewed any marks of inflammation. The internal surface of the fauces and œsophagus exhibited very faint traces of inflammatory action. The brain was minutely examined by Dr. Johnson, who favoured me with the following minutes; viz.

" Dura mater not particularly adherent to the cranium. Vessels of the pia mater gorged with blood. The tunica arachnoides inflamed in some places, and thickened all over the hemispheres. Great effusion of water under the tunica arachnoides, filling all the convolutions of the brain. On tearing up any portion of the pia mater from the substance of the brain, the vessels seemed to break from turgescence rather than inflammation. The substance of the brain itself unusually, indeed remarkably firm. Lateral ventricles full of water, containing, perhaps, two ounces, or more. The pia mater lining the parietes of the ventricles, in some places, finely injected; in others blanched. The plexus choroides blanched in the effusion. All the parts in the interior of the brain were firm, and easily demonstrated. On turning up the anterior lobe, the neurilema of the optic nerves was inflamed, and large turgid vessels were seen running along them. The tunica arachnoides here was much thickened, and quite opake. The pia mater covering the *base* of the brain was considerably inflamed, and finely injected. There was also effusion here. The tentorium appeared exceedingly tense, and when slit open, the cerebellum actually started. The coverings of the base of the cerebellum, pons Varolii, medulla oblongata, &c. formed one crust of intense inflammation. A slender knife was passed as far as possible down the vertebral canal, and the medulla spinalis cut there. The same crust of inflammation was continued along the spinal marrow, and appeared, if possible, more intense than on the medulla oblongata. There could, therefore, be no manner of doubt that the whole envelope of the spinal cord was covered with inflammation. A considerable quantity of bloody serum ran from the vertebral canal for two hours after the cerebral dissection was discontinued."

*(Signed.)* "J. JOHNSON, M.D."

Perhaps it might be useful to divide the period, from the time that a person is bitten by a rabid animal till his death, into four stages.—*The first*, from the infliction of the bite to any given time before pain in the wound commences: the cure then might be attempted by excision and cauterization.

*The second*, from the commencement of pain to the time that it has nearly reached the top of the limb. At this period, I should apprehend that amputation is the only means likely to prevent constitutional symptoms from supervening.

*The third stage*, from the commencement of pyrexia, however slight, to a state of collapse. Here, I suppose, from the appearances exhibited on dissection, that venæsection *to a great extent*, blisters to the head and spine, and the exhibition of enormous quantities of the hydrargyri submurias, would afford the most probable chance of effecting a cure.

R. WEBSTER.

Portsmouth, September 2, 1817.

---

The almost total absence of all *pyrexial* symptoms, while the base of the brain, origins of the nerves, and coverings of the medulla oblongata, and medulla spinalis, were in such a state of inflammation, is a circumstance well calculated to call forth reflections, and direct the attention of the pathologist to the investigation of *nervous and spasmodic* diseases, through the aid of morbid anatomy. Never was *spasm* or *nervous* irritation more marked than here. Never did death take place with less pyrexia; and yet intense inflammation of the basis cerebri and spinal neurilema, with effusion into the convolutions, ventricles, vertebral canal, &c. was discovered on dissection! Surely this case is calculated to let in a flood of light on *nervous* and *spasmodic* diseases. Vide *Dr. Moulson's paper in this Journal.* EDITORS.

